# Effect of Laser Remelting on Friction-Wear Behaviors of Cold Sprayed Al Coatings in 3.5% NaCl Solution

**DOI:** 10.3390/ma11020283

**Published:** 2018-02-11

**Authors:** Zhang Jing, Kong Dejun

**Affiliations:** School of Mechanical Engineering, Changzhou University, Changzhou 213164, China; 16105527@smail.cczu.edu.cn

**Keywords:** cold spraying, Al coating, laser remelting (LR), friction and wear, corrosion wear, wear mechanism

## Abstract

A cold sprayed Al coating on S355 structural steel was processed using a laser remelting (LR). The surface and cross-section morphologies, chemical compositions, and phases of as-obtained Al coating before and after LR were analyzed using a scanning electronic microscope (SEM), energy dispersive spectrometer (EDS), and X-ray diffractometer (XRD), respectively, and their hardness was measured using a micro-hardness tester. The friction-wear behaviors of Al coating before and after LR in 3.5% NaCl solution were conducted to simulate the sand and gravel scouring on its surface in seawater, the effects of wear loads and speeds on the tribological properties of Al coating were analyzed, and the wear mechanisms under different wear loads and speeds were also discussed. The results show that the Al coating after LR is primarily composed of an Al phase and its hardness is 104.66 HV, increasing 54.70 HV than the cold sprayed Al coating. The average coefficient of friction (COF) of cold sprayed Al coating at the wear load of 0.5, 1.0 and 1.5 N is 0.285, 0.239, and 0.435, respectively, while that after LR is 0.243, 0.227, and 0.327, respectively, decreased by 14.73%, 5.02% and 24.83% compared to the cold sprayed Al coating. The wear rate of cold sprayed Al coating at the wear load of 0.5, 1.0 and 1.5 N is 1.60 × 10^−4^, 2.36 × 10^−4^, and 2.40 × 10^−4^ mm^3^/m·N, respectively, while that after LR is 1.59 × 10^−4^, 1.70 × 10^−4^, and 1.94 × 10^–4^ mm^3^/m·N, respectively, decreased by 1%, 32%, and 23%, respectively, indicating that LR has high anti-friction performance. Under the wear load action of 1.0 N, the average COF of laser remelted Al coating at the wear speeds of 300, 400 and 500 times/min is 0.294, 0.279, and 0.239, respectively, and the corresponding wear rate is 1.06 × 10^−4^, 1.24 × 10^−4^, and 1.70 × 10^−4^ mm^3^/m·N, respectively. The wear mechanism of cold sprayed Al coating is primarily corrosion wear at the loads of 0.5 and 1.0 N, and that at the load of 1.5 N is abrasive wear and fatigue wear; while that after LR is abrasive wear and fatigue wear, with no corrosion wear, showing that LR improves its corrosion and wear resistance.

## 1. Introduction

As a structural steel, S355 structural steel has the characteristics of high strength, plasticity and impact toughness etc. [1,2] and is mainly used in manufacturing of offshore platforms. When used in the marine environment for a long time, it is prone to corrosion and being worn by small sandstone; Al coating is generally used for protection. Cold spraying is a fabrication method of Al coating with plastic deformed Al powders impacted by high speed gas to combine with the substrate [3,4,5,6,7,8], because the cold sprayed Al coating presents a typical layered structure with micro-porosity, it is difficult to adapt to the harsh environment, which limits its scope of application and service life on offshore platforms [9,10]. In order to improve the properties of cold sprayed Al coating, the surface modification of Al coating is processed using a post-processing technology. Laser remelting (LR) is used to improve the hardness, wear resistance and corrosion resistance of Al coating, which has little effect on the surface roughness and the size of the coatings [11,12,13]. At present, LR has been used in post-processing to improve the performance of Al coating [14], and has achieved some results. Wang et al. fabricated a MCrAlY coating on Ti–Al alloy using plasma spraying: the lamellar micro-structure disappeared and the density increased and other defects disappeared after LR [15]; Astaritaa et al. used LR to modify the cold-sprayed Ti coating: the remelted zone (RZ), heat affected zone (HAZ) and base zone (BZ) were formed after LR [16]; Yao J et al. analyzed the beneficial effects of laser irradiation on the deposition process of cold-sprayed diamond and Ni60 composite coatings [17]. Almangour B et al. analyzed the scanning strategies for texture and anisotropy tailoring during selective laser melting of TiC/316L stainless steel nanocomposites and selective laser melting of TiC reinforced 316L stainless steel matrix nanocomposites: influencing the starting TiC particle size and volume content [18,19]. LR can effectively eliminate the layered structure and porosity of cold sprayed Al coating, and forms a uniform and compact structure, which is an effective way to improve the properties and expand the application range of cold sprayed Al coating [20,21,22]. In this study, a cold sprayed Al coating on S355 structural steel was processed using an LR. The friction-wear behaviors of Al coating were investigated to simulate the sand wear in seawater, which provided an experimental basis for the Al coating protection of S355 steel on offshore platforms.

## 2. Experimental

The substrate was European standard S355 structural steel with the chemical components (wt %) C 0.17, Si 0.55, Mn 0.94, P 0.035, Cr 0.065, S 0.035, Ni 0.065, Mo 0.30 and Zr 0.15; the rest was Fe. The spraying process was as follows: mechanical polishing → chemical washing to remove oil → rust removal → rinsing and drying → cold sprayed Al coating → cooling → cleaning surface. The cold sprayed powder was pure Al particles with diameters 20–45 μm. The cold spraying test was conducted on Kinetiks 4000-type cold spraying equipment, and spraying parameters were as follows: pressure of 3.5 MPa, temperature of 400 °C, the distance between spraying gun and sample was 40 mm, gun speed of 200 mm/s, powder feeding of 1 L/min. After the cold spraying test, the obtained Al coating was sealed using a DIAMANT Dichtol WFT (1532) type sealing agent. The cold sprayed Al coating was remelted using a 2000W all-solid fiber coupling transmission laser spraying system; the technological parameters of LR are shown in Table 1. After the LR test, the surface and cross-section morphologies and chemical elements of Al coating were analyzed using a JSM-6360LA type SEM (NEC Electronics Corporation, Tokyo, Japan) and its configured EDS (NEC Electronics Corporation, Tokyo, Japan), respectively, and its phases were analyzed using a D/max2500 PC type XRD (Rigaku Corporation, Tokyo, Japan). The hardness of Al coating before and after LR was measured using an HMV-2T (Hardness Micro Vickers-2 Transformation) type Vickers micro-hardness tester (Shimadzu Enterprise Management (China) Co., Ltd., Shanghai, China).

Before the friction-wear test, the samples were immersed in 3.5% NaCl solution for 120 min. The friction-wear test was carried out on a CFT-I type reciprocating friction-wear tester (Lanzhou Zhongke Kaihua Science and Technology Development Co. Ltd., Lanzhou, China), with a measurement accuracy of COF of 0.2% FS (full scale), a depth measurement range of ±0.5 mm, and accuracy of 0.1 μm. The Al coating was immersed in 3.5% NaCl solution to investigate the effects of wear loads and wear speeds on the friction-wear behaviors of Al coating before and after LR, the friction-pair was a Si_3_N_4_ ceramic ball with a diameter of 3.5 mm, a wear distance of 5 mm, a test time of 2 h, a respective load of 0.5, 1.0, and 1.5 N, and rotation speed of 500 times/min. After the above wear test, the optimized wear load was selected to compare the friction-wear behaviors at rotation speeds of 300, 400, and 500 times/min. The morphologies, chemical element distributions and phases of worn tracks were analyzed using a SEM, EDS, and XRD, respectively, and the wear profiles of worn tracks were measured using a VHX–700FC type super–depth three–dimensional microscopic system (Keyence Corporation, Osaka, Japan).

## 3. Analysis and Discussion of Results

### 3.1. Characterization of Al Coatings

#### 3.1.1. Morphologies of Al Coating Surfaces and Cross Sections

**Figure 1:**
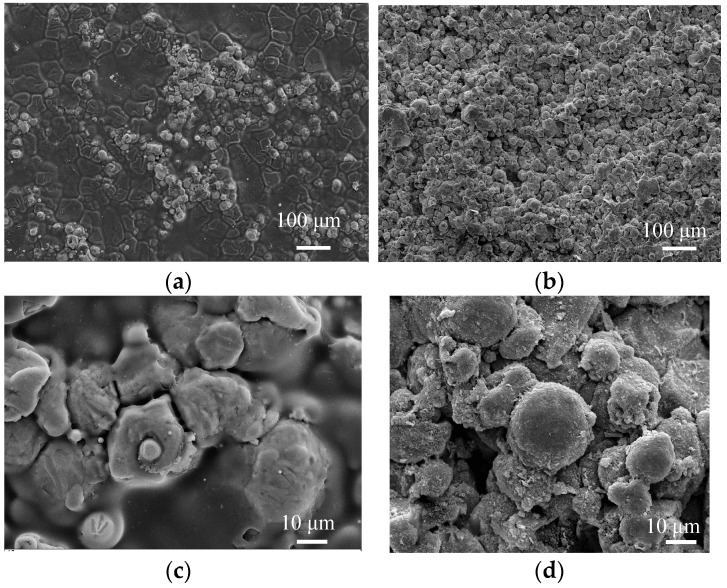

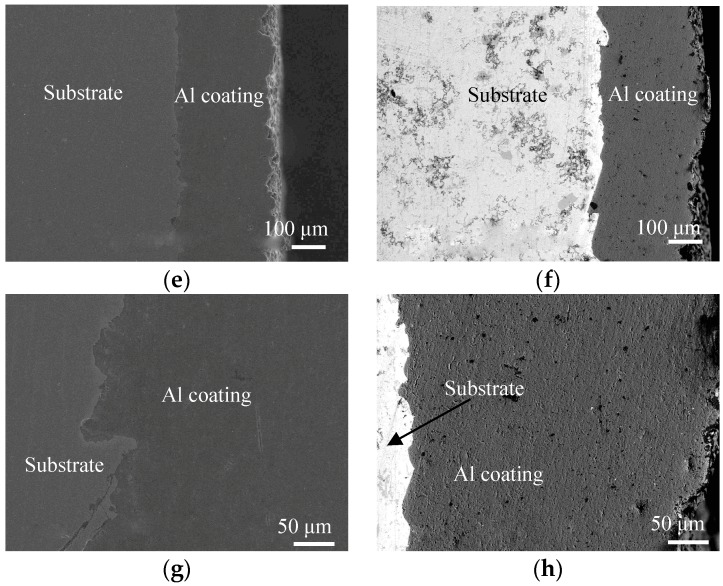
Surface and cross section morphologies of cold sprayed Al coating before and after laser remelting (LR). (**a**) Low-magnified surface morphology before LR; (**b**) Low-magnified surface morphology after LR; (**c**) High-magnified surface morphology before LR; (**d**) High-magnified surface morphology after LR; (**e**) Low-magnified cross-section morphology before LR; (**f**) Low-magnified cross-section morphology before LR; (**g**) High-magnified cross section morphology before LR; (**h**) High-magnified cross section morphology after LR.

The surface and cross-section morphology of cold sprayed Al coating before and after LR is shown in Figure 1a,b. The cold sprayed Al particles appeared as an obvious deformation when impacting on the substrate at high speed, which were distributed on the substrate as flat strips, and they were mechanically embedded with the substrate [23,24]. The Al particles were closely combined, and there was a clear interface between the particles, with no obvious porosity on the Al coating surface. The morphology of laser-remelted Al coating remained intact; its micro-structure was changed due to the laser thermal effect. After LR, the flat stripe and mosaic structure disappeared, the Al particles were fine and uniform, and the Al coating was almost completely compact. The porosity and inclusions were reduced greatly, and the remelted layer was obtained without cracks, porosity and other defects.

Figure 1c,d shows the highly-magnified morphology of cold sprayed Al coating surface before and after LR. The Al particles on the Al coating surface before LR were not tamped by the subsequent particles, the spherical powders remained intact, and the Al particles were obviously found on the Al coating surface. Compared with the cold sprayed Al coating, the profiles of Al particles were gradually desalinated after LR, which was due to the appearance of melted chip-shaped products in the LR process, which indicated that a little amount of dissolution at the edge of Al particles; however, the overall shape of Al particles changed little.

Figure 1e,f shows the low-magnified cross-section morphology of cold sprayed Al coating before and after LR. Before LR, the Al coating was mechanically bonded with the substrate and the separation line of Al coating-substrate was obviously coherent, with no obvious defects. After LR, the cross-section of Al coating was smoother, the loose structure, fine porosity and micro-cracks were gradually eliminated with the laser powers increasing, and the grains of Al coating were refined. The porosity was unevenly distributed on the cross-section of Al coating, and its density was high.

Figure 1g,h shows the highly-magnified morphology of Al coating cross-section before and after LR. It can be seen that there were isolated porosity and cracks on the cross-section of Al coating before LR: due to the impact of the high speed in the cold spraying process, the Al particles with the un-melted state produced plastic deformation during the stacking process, and the grain boundaries remained among them. After LR, the Al coating was heated and cooled down very quickly during the LR process, which improved the micro-structure and eliminated the grain boundary between the Al particles. The porosity was accumulated near the substrate, this was because the cooling rate of Al coating near the coating surface was greater than that of Al coating near the substrate during the LR process, the inner gas was not discharged in time, and the porosity was formed near the substrate.

#### 3.1.2. XRD Analysis

**Figure 2:**
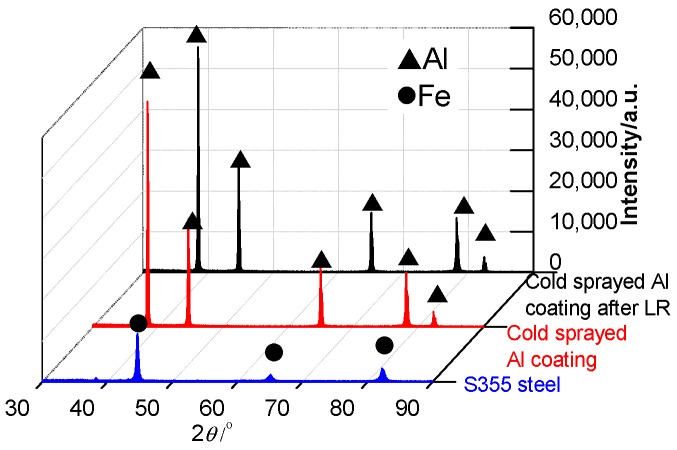
XRD analysis of cold sprayed Al coatings before and after LR.

Figure 2 shows the XRD spectra of S355 steel and cold sprayed Al coating before and after LR. The phase of S355 steel was Fe, while that of cold sprayed Al coating was Al: no oxides were detected. After LR, the Al coating phase was the same with that before LR and the Al coating maintained the original phase and showed no phase transition; the Al particles were not oxidized after LR. This reason was shown as follows: (a) the Al powder phase stably existed due to low temperature during the cold spraying process; (b) although the bonding method of Al coating was changed from mechanical bonding to metallurgical bonding, no new phase was precipitated in the rapid cooling process, the original micro-structure of Al was inherited. Therefore, there was no phase transformation during the LR process.

#### 3.1.3. Hardness Analysis

The surface hardness of cold sprayed Al coating before and after laser remelting is shown in Table 2. The average hardness of Al coating before LR was 49.96 HV, while that of Al coating after LR was 104.66 HV: an increase of 109% compared to before LR, which was related to the working hardening and thermal action during the LR test. In addition, the porosity of laser remelted Al coating was greatly reduced to almost completely compaction, which caused hardness of Al coating increase.

### 3.2. COFs and Wear Profiles

#### 3.2.1. Effect of Load on Friction-Wear

**Figure 3:**
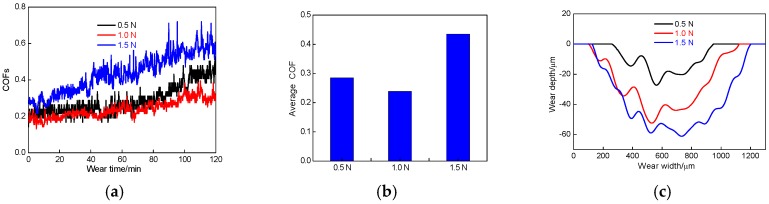
Coefficients of friction (COFs) vs. wear time and wear profiles of cold sprayed Al coatings before LR. (**a**) COFs vs. wear time; (**b**) Average COFs; (**c**) Wear profiles.

Figure 3a shows the COFs of cold sprayed Al coating in 3.5% NaCl solution at the loads of 0.5, 1.0 and 1.5 N (wear speed of 500 times/min). The COFs at the loads of 0.5, 1.0 and 1.5 N increased with the wear time increasing, which was related to the principle of cold sprayed Al coating. Under high-speed impact, the Al particles were deposited with severe plastic deformation: the first deposited particles were subjected to the impact of subsequent particles to form a micro-forging combination, which caused the bonding inside the Al coating to become stronger. According to the cold spraying mechanism, the density of cold sprayed Al coating increased gradually from the Al coating surface to the substrate. When the Al coating surface was peeled off, the ceramic ball gradually contacted with the higher hardness of Al coating, which caused the COF increase.

The average COF of Al coating at the loads of 0.5, 1 and 1.5 N was 0.285, 0.239, and 0.435, respectively, and the standard deviation for COF of cold sprayed Al coating was 8.05 × 10^−2^, 4.69 × 10^−2^ and 10.49 × 10^−2^, respectively; among them, the average COF and standard deviation for COF at the load of 1 N were the smallest, showing the better friction performance, as shown in Figure 3b. The friction pair at the load of 0.5 N moved on the shallow Al coating surface: the pits and peaks on the Al coating surface hindered the sliding of the ceramic ball. The Al coating surface was fluffy, which led to higher COFs. The friction layer at the load of 1 N was smooth in the Al coating depth. Because of actual contact stresses increasing, the Al coating was easily plowed and the pit was smooth, so the COFs were kept lower and the curve was stable. The Al coating surface at the load of 1.5 N was hardened due to plastic deformation, the friction pairs easily was stuck and decreased the shearing resistance [25,26,27], which led to large COFs.

The wear profile of Al coating in NaCl solution under different loads is shown in Figure 3c. The wear rate was calculated by the following equation:

(1) $$W=\frac{V}{S\times P}$$

where *V* was the wear losses of the coating; *S* was the sliding distance; and *P* was the normal load applied.

The wear rates of Al coatings at the loads of 0.5, 1.0 and 1.5 N were 1.60 × 10^−4^, 2.36 × 10^−4^, and 2.40 × 10^−4^ mm^3^/m·N, respectively. The wear rates increased with the wear loads increasing, which was due to the shearing and compressive stress at the large wear loads was bonded among the Al particles, which increased with the wear loads increasing, and the falling probability of Al particles increased gradually. The fell particles were used as abrasive particles to wear the Al coating, which reduced the wear resistance and increased the wear rate.

The laser-remelted Al coating had regional characteristics because the laser beam spot was scanned at the certain speed on the Al coating: its surface was first heated and then the heating was transferred from the surface through the heat conduction into the inner coating, which caused an uneven distribution of temperature in the molten pool and produced the uneven mass-transfer in the molten pool. On the central region of the laser beam near the substrate, the pool temperature was very high, while the substrate temperature was relatively low due to the temperature gradient; the temperature-distribution and solidification rate were different on the regions of molten pool, which led to different micro-structures on the different regions of the molten layer [28,29]. The friction-wear properties of surface layer of 0~70 μm on the cold sprayed Al coating after LR were mainly investigated, which is considered the surface layer as the remelted layer of Al coating.

**Figure 4:**
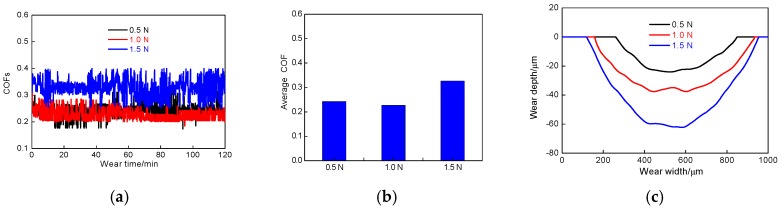
COFs vs. wear time and wear profiles of cold sprayed Al coatings after LR. (**a**) COFs vs. wear time; (**b**) Average COFs; (**c**) Wear profiles.

Figure 4a shows the COFs of laser-remelted Al coating at the wear loads of 0.5, 1.0 and 1.5 N in 3.5% NaCl solution (friction rate of 500 times/min), respectively. It can be seen that the COFs of laser-remelted Al coating fluctuated up and down in a certain range without a large overall upward or downward trend: this was because the Al coating after LR was affected by many factors, such as self-diffusion and gravity. Compared with the Al coating before LR, the structure after LR was very compact and uniform, the hardness was generally improved, and the hardness gradient on worn track disappeared, so the COFs fluctuated in a fixed range. The brittleness of worn track on the Al coating surface increased after LR. Because of the large shearing force and the fact that the bonding of the Al coating was unstable, some Al particles was flaked from the Al coating, and the peeled Al particles became new abrasive particles to participate in the wear test on the worn track. Due to the instability of new Al particles, which were easily dispersed into the NaCl solution during the wear test, the COFs were made to fluctuate up and down.

As shown in Figure 4b, the average COFs of cold sprayed Al coating after LR were 0.243, 0.227 and 0.327, and the standard deviation for COF of cold sprayed Al coating was 2.66 × 10^−2^, 2.25 × 10^−2^ and 3.63 × 10^−2^, respectively; compared to that before LR, these values were generally lower, which was due to the increasing of Al coating hardness after LR. The depth of micro-convex or abrasive particles pressed into the Al coating surface decreased and the friction resistance reduced, which caused the coefficient decrease.

After LR, the profiles of worn tracks are shown in Figure 4c. The wear rates of Al coatings at the wear loads of 0.5, 1.0 and 1.5 N were 1.59 × 10^−4^, 1.70 × 10^−4^ and 1.94 × 10^−4^ mm^3^/m·N, respectively, indicating that the wear depth increased with the wear loads increasing. Compared with the Al coating before LR, the worn tracks of Al coating were smoother after LR, which was due to the dense and high hardness of Al coating after LR, the finer and smoother abrasive particles were flake, the worn track was a typical smooth pit shape.

#### 3.2.2. Effect of Wear Speed on Friction-Wear

**Figure 5:**
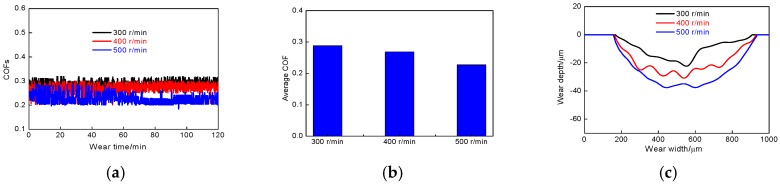
COFs vs. wear time and wear profiles of cold sprayed Al coatings after LR at different wear speeds. (**a**) COFs vs. wear time; (**b**) Average COFs; (**c**) Wear profiles.

It can be seen from the above that the average COF of laser-remelted Al coating at the load of 1.0 N was the smallest, indicating that the Al coating had better friction performance at the load. The friction-wear performance of cold sprayed Al coating in 3.5% NaCl solution was analyzed by setting the load at 1.0 N; meanwhile, changing the speed of friction pair. Figure 5a shows the COFs vs. different wear speeds. Figure 5b shows the average COFs of Al coating at the different wear speeds. The average COFs at the wear speeds of 300, 400 and 500 times/min were 0.294, 0.279 and, 0.239, and the standard deviation for COF of cold sprayed Al coating was 1.62 × 10^−2^, 2.20 × 10^−2^ and 2.25 × 10^−2^, respectively. The above-average COFs were less than 0.3 at the wear speeds of 300, 400 and 500 times/min, indicating that the Al coating had better friction performance after LR. The COFs became smaller with the wear speed increasing, and the variation of COFs tended to be more stable during the friction test, this was because when the wear speed was small, the surface temperature of friction pair in NaCl solution was lower, and the ceramic ball was easily cut and extruded the Al coating, so the wear amount was relatively low. However, the plastic deformation resistance of the friction pair was large: the plastic deformation resistance between the friction surfaces was mainly due to the interaction between the small convex peaks of contact surfaces between the friction surfaces, so the COFs were large when the friction resistance between the friction surfaces was larger. With the friction speeds increasing, the COFs of friction pair were reduced effectively under the synergistic action of water lubrication and the friction contact area increased.

From Figure 5c, it can be seen that the wear pits were smooth and few irregular pits appeared. The maximum depth of corresponding pits at the wear speeds of 300, 400 and 500 times/min was 23, 30 and 37 μm, respectively. The corresponding wear rate was 1.06 × 10^−4^, 1.24 × 10^−4^, and 1.70 × 10^−4^ mm^3^/m·N, indicating that the wear rates increased with the wear speeds increasing, the wear of fine abrasive particles on the worn track were accelerated to increase the wear amount.

### 3.3. Plane Scanning Analysis of Worn Tracks

**Figure 6:**
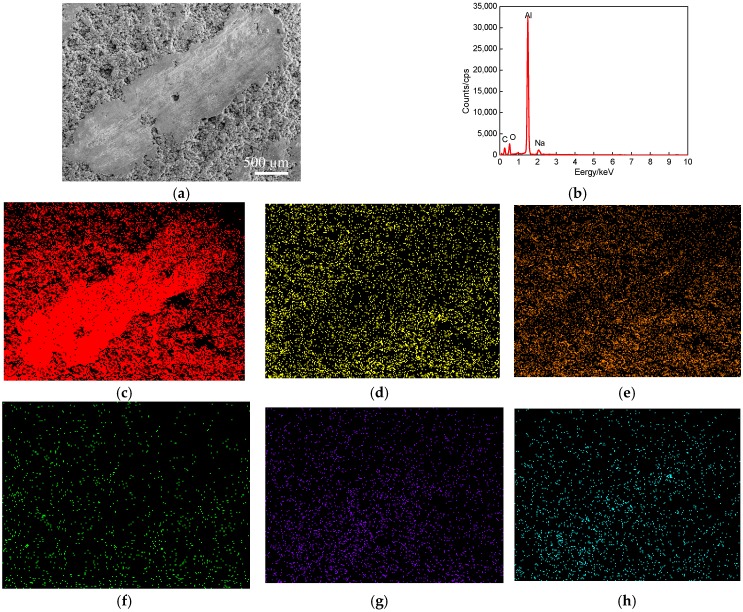
Plane scan analysis of worn track at wear speed of 300 times/min (wear load of 1 N). (**a**) Plane scanned position; (**b**) Result of plane scan analysis; (**c**) Al content; (**d**) C content; (**e**) O content; (**f**) Fe content; (**g**) Na content; (**h**) Cl content.

The plane scanned position of a worn track at the wear speed of 300 times/min (wear load of 1.0 N) is shown in Figure 6a. The plane scanning result is shown in Figure 6b, and the mass fractions of chemical elements (mass, %) were C 65.14, O 23.90, Na 0.07, Al 10.45, and Cl 0.35; the corresponding atomic fractions (at, %) were Al 27.86, C 41.44, O 20.29, Fe 0.21, Na 0.10 and Cl 0.10. The sealing agent on the cold sprayed Al coating surface produced some volatilization under the high-temperature remelting condition; the Al atom-rich zones existed on the worn track, as shown in Figure 6c. The C and O were uniformly distributed, indicating that C and O appeared as the form of compounds on the Al coating, which were the remains of sealing agents, as shown in Figure 6d,e. The C and O appeared as atom-poor zones on the worn track: this was because the remains of sealing agent were peeled off from the Al coating. The Fe was massively distributed on the laser remelted Al coating, as shown in Figure 6f, which was caused by the thermal diffusion of Fe in the substrate into the side of Al coating in the LR process. The Na and Cl content were very little, which were the residues came from NaCl solution, as shown in Figure 6g,h.

**Figure 7:**
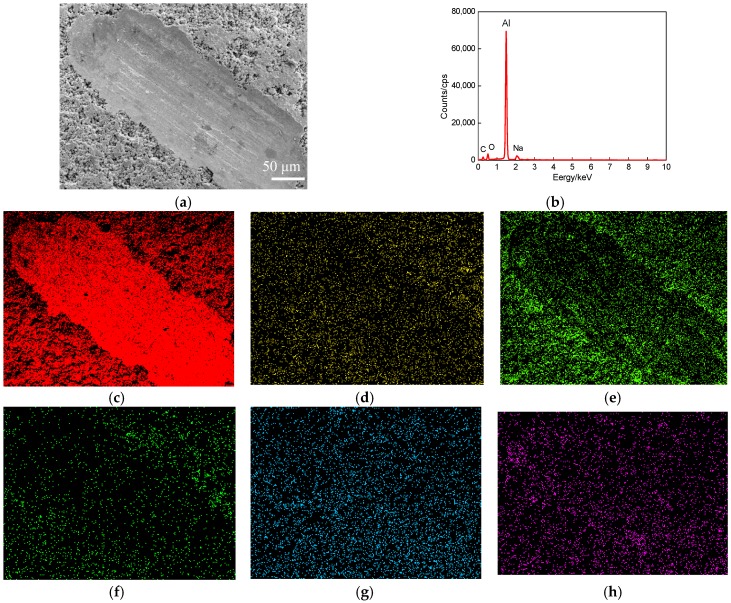
Plane scan analysis of worn track at wear speed of 400 times/min (wear load of 1 N). (**a**) Plane scanned position; (**b**) Result of plane scan analysis; (**c**) Al content; (**d**) C content; (**e**) O content; (**f**) Fe content; (**g**) Na content; (**h**) Cl content.

Figure 7a shows the plane scanned position of worn track at the wear speed of 400 times/min. The plane scanned result is shown in Figure 7b, the mass fractions of chemical elements (mass, %): Al 52.74, C 20.59, O 25.48, Fe 0.39, Na 0.27 and Cl 0.53, and the corresponding atomic fractions (at, %): Al 37.02, C 32.71, O 29.72, Fe 0.13, Na 0.22 and Cl 0.2. The Al content increased by 18% than that at the wear speed of 300 times/min, as shown in Figure 7c. The other elements changed little, the plane scan distribution was the same as that at the wear speed of 300 times/min, as shown in Figure 7d–g.

**Figure 8:**
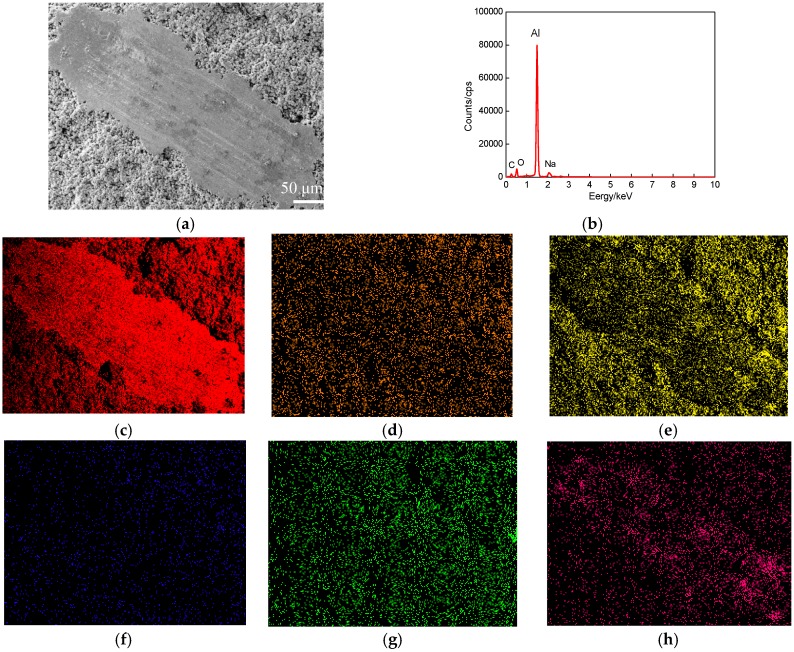
Plane scan analysis of worn track at wear speed of 500 times/min (wear load of 1 N). (**a**) Plane scanned position; (**b**) Result of plane scan analysis; (**c**) Al content; (**d**) C content; (**e**) O content; (**f**) Fe content; (**g**) Na content; (**h**) Cl content.

Figure 8a shows the plane scanned position of a worn track at the wear speed of 500 times/min. The plane scanned result is shown in Figure 8b, the mass fractions of chemical elements (mass, %) were Al 55.22, C 22.49, O 20.95, Fe 0.79, Na 0.21 and Cl 0.34, and the corresponding atomic fractions (at, %) were Al 38.88, C 35.51, O 24.99, Fe 0.27, Na 0.17 and Cl 0.18. The Al increased by 5% compared to that at the wear speed of 400 times/min, as shown in Figure 8c. The other elements changed little: their plane scanning distributions were the same as those at the wear speeds of 300 and 400 times/min, as shown in Figure 8d–g.

From the above analyses, the Al content on the worn tracks increased as the wear speeds increased, this was because the increasing of wear speeds resulted in increasing the abrasive wear to widen the widths of worn tracks, and the Al content increased correspondingly.

### 3.4. Wear Mechanism

**Figure 9:**
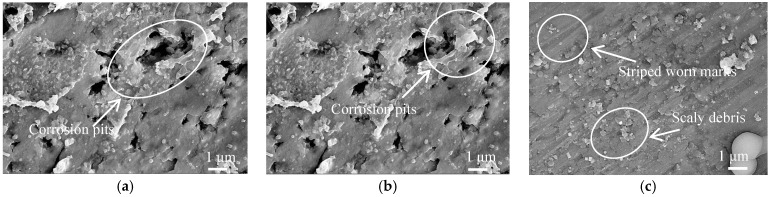
Morphologies of worn track on cold sprayed Al coatings before LR. (**a**) Wear load of 0.5 N; (**b**) Wear load of 1.0 N; (**c**) Wear load of 1.5 N.

A large number of corrosion pits appeared on the worn track at the wear load of 0.5 N, and rough and loose corrosion products were formed on the Al coating surface, as shown in Figure 9a. The friction pairs were contacted on the Al coating surface, and the characteristics of cold spraying resulted in the loose and porosity on the Al coating surface at the low wear loads. The Cl^−^ in 3.5% NaCl solution entered the Al coating internally through the porosity and corrosion, which caused the Al on the Al coating surface to become a soluble salt. Because of the high conductivity of Cl^−^, the cation transfer speed was improved, which accelerated the corrosion current flow and decreased the corrosion rate.

The corrosion mechanism of Al coating was primarily electrochemical corrosion. The electrode reactions are shown as follows.

On the anode, the reaction was

(2) $$\mathrm{Al}\to {\mathrm{Al}}^{3}{}^{+}{+3e}^{-}$$

On the cathode, the reaction was

(3) $$O_{2}{+H}_{2}{O+4e}^{-}\to {4\mathrm{OH}}^{-}$$

(4) $${\mathrm{Al}}^{3+}{+3\mathrm{OH}}^{-}\to {\mathrm{Al}}_{2}{\left(\mathrm{OH}\right)}_{3}$$

(5) $${2\mathrm{Al}}_{2}{\left(\mathrm{OH}\right)}_{3}\to {\mathrm{Al}}_{2}O_{3}{+3H}_{2}O$$

(6) $${\mathrm{Al}}_{2}{\left(\mathrm{OH}\right)}_{3}{+\mathrm{Cl}}^{-}\to {\mathrm{Al}(\mathrm{OH})}_{2}{\mathrm{Cl}+\mathrm{OH}}^{-}$$

(7) $${\mathrm{Al}(\mathrm{OH})}_{2}{\mathrm{Cl}}^{-}{+\mathrm{Cl}}^{-}\to {\mathrm{Al}(\mathrm{OH})\mathrm{Cl}}_{2}{+\mathrm{OH}}^{-}$$

(8) $${\mathrm{Al}(\mathrm{OH})\mathrm{Cl}}_{2}{+\mathrm{Cl}}^{-}\to {\mathrm{AlCl}}_{3}{+\mathrm{OH}}^{-}$$

During the corrosion reaction, the Al^3+^ concentration in Equation (2) increased, which reacted with the OH^−^ in Equation (3) and produced the white precipitate of Al(OH)_3_ in Equation (4) after corrosion reaction. The precipitate underwent a secondary reaction to produce the Al_2_O_3_ in Equation (5) [30]. The rest of the Al_2_(OH)_3_ reacted with the Cl^−^ to produce the Al(OH)_2_Cl in Equation (6), showing an obvious corrosion of the Al coating. The Cl^−^ adhered to the Al(OH)_2_Cl^−^ on the Al coating to produce the Al(OH)Cl_2_ in Equation (7), resulting in a thinner oxidation film and a bared Al coating. The Al(OH)Cl_2_ continuously reacted with the Cl^−^ to produce the AlCl_3_ in Equation (8) [31].

The hardness of the friction pair increased at the wear load of 1.0 N, while the porosity rate and corrosion degree decreased, as shown in Figure 9b; only a small number of isolated corrosion pits existed on the corrosion area. It can be seen that the wear mechanism was corrosion wear at the wear loads of 0.5 and 1.0 N. There were strip-worn tracks and small scaly flakes on the worn tracks at the wear load of 1.5 N, as shown in Figure 9c. The new abrasive particles were always produced in the wear process, and ground the Al coating to form the striped furrows on the Al coating surface, causing the Al particles on the Al coating surface to fall off; the wear mechanism was abrasive wear [32]. Due to the stress concentration at the contact point between the Al coating surface and the ceramic ball, the fatigue cracks and worn debris appeared under the action of circular stress, and such cracks mainly appeared in the form of scaly flakes, at the same time, the hardness of worn track increased, resulting in fatigue wear [33,34,35]. There were flaky debris appearing on the worn track, and the hardness on the worn track was 71.10 HV, larger than the Al coating surface, which was the specific feature of fatigue wear. The wear mechanism of cold sprayed Al coating at the load of 1.5 N was abrasive wear + fatigue wear.

**Figure 10:**
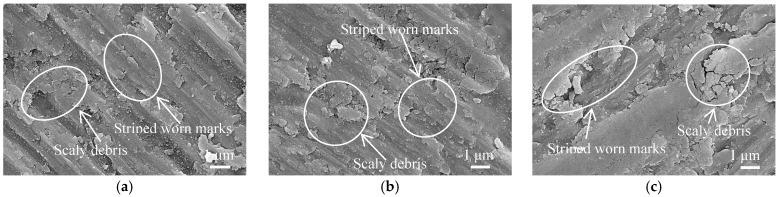
Morphologies of worn track on cold sprayed Al coatings after LR. (**a**) Wear load of 0.5 N; (**b**) Wear load of 1.0 N; (**c**) Wear load of 1.5 N.

The cold-sprayed Al coating surface was denser after LR, and the porosity and defects reduced, which prevented the adsorption and internal diffusion of Cl^−^ and slowed down the corrosion rate. The hardness of worn tracks at the loads of 0.5, 1.0 and 1.5 N was 152.12, 163.27, and 168.42 HV, respectively, which was much higher than that of Al coating surface. As shown in Figure 10a–c, no corrosion pits and corrosion products were found, but the scaly worn debris appeared on the worn tracks, which were corresponded to the characteristics of fatigue wear. The stripe track appeared caused by abrasive wear on the worn track, which was concluded that the Al coating undergone the abrasive wear. The wear mechanism of laser remelted Al coating at the loads of 0.5, 1.0 and 1.5 N was abrasive wear + fatigue wear.

## 4. Conclusions

(1) The average hardness of cold sprayed Al coating was 49.96 HV, while that after LR was 104.66 HV, increasing by 109.5% compared to that before LR.
(2) The average COFs of cold sprayed Al coatings at the wear loads of 1, 3 and 5 N were 0.285, 0.239, and 0.435, respectively, and the corresponding wear rates were 1.60 × 10^−4^, 2.36 × 10^−4^ and 2.40 × 10^−4^ mm^3^/m·N, respectively; while those after LR were 0.243, 0.227, and 0.327, respectively, and the corresponding wear rates are 1.59 × 10^−4^, 1.70 × 10^−4^ and 1.94 × 10^−4^ mm^3^/m·N, respectively, showing that the friction-wear properties of Al coatings are improved after LR.
(3) The average COFs of Al coatings at the wear speeds of 300, 400 and 500 times/min (wear load of 1.0 N) were 0.294, 0.279, and 0.239, respectively, and the corresponding wear rates were 1.06 × 10^−4^, 1.24 × 10^−4^, and 1.70 × 10^−4^ mm^3^/m·N, showing the lower COFs and wear rates.
(4) The wear mechanism of cold sprayed Al coating in 3.5% NaCl solution is corrosion wear under 0.5 and 1.0 N load, which is abrasive wear + fatigue wear under 1.5 N; while that after LR that is abrasive wear + fatigue wear, with no corrosion wear, showing that LR reduces the porosity of Al coating and improve its corrosion wear resistance.

## Figures and Tables

**Table 1:** Technological parameters of cold sprayed Al coatings.

Item	Value
Spray distance/mm	250
Spot diameter/mm	5
laser power/W	800
Speed of argon gas/L/min	8

**Table 2:** Hardness of cold sprayed Al coating before and after LR.

Status	Value/HV					Average Value/HV
Before LR	47.2	49.3	52.3	47.7	53.3	49.96
After LR	101.4	104.6	100.5	106.2	110.6	104.66
